# Editorial: Computational chemogenomics: In silico tools in pharmacological research and drug discovery

**DOI:** 10.3389/fphar.2023.1150869

**Published:** 2023-03-17

**Authors:** Norberto Sánchez-Cruz, Eli Fernandez-de Gortari, José L. Medina-Franco

**Affiliations:** ^1^ Instituto de Química, Unidad Mérida, Universidad Nacional Autónoma de México, Ucú, México; ^2^ Instituto de Investigaciones en Matemáticas Aplicadas y en Sistemas, Unidad Académica del Estado de Yucatán, Universidad Nacional Autónoma de México, Mérida, México; ^3^ International Iberian Nanotechnology Laboratory (INL), Braga, Portugal; ^4^ DIFACQUIM Research Group, Department of Pharmacy, School of Chemistry, Universidad Nacional Autónoma de Mexico, Mexico City, Mexico

**Keywords:** cheminformatics, computational chemistry, molecular dynamics, artificial intelligence, machine learning, molecular docking, virtual screening, toxicology

Chemogenomics aims towards the systematic identification of small molecules that interact with protein targets and modulate their function (Quinlan et al., 2021). This research field is a crucial discipline in pharmacological research and drug discovery, as it allows the identification of novel bioactive compounds and therapeutic targets, as well as the elucidation of the mechanism of action of known drugs. In principle, the final goal of chemogenomics is identifying small molecules that can interact with any biological target. However, considering the number of existing small molecules and biological targets, this task is basically impossible to achieve experimentally. Developments in computer science-related disciplines, such as cheminformatics, molecular modelling, and artificial intelligence (AI) have made possible the *in silico* analysis of millions of potential interactions between small molecules and biological targets, prioritizing on a rational basis the experimental tests to be performed, reducing with that the time and costs associated with them. These computational approaches represent the toolbox of computational chemogenomics (Mestres, 2004; Jacoby et al., 2018).

Methods from computational chemogenomics have become crucial in pharmacological research and drug discovery. Advances in computer science and AI, as well as the growing availability of experimental data, have opened the door to the development and refinement of new computational models. These models require thorough validation and dissemination within the scientific community. The present Research Topic brings together experts that discuss, in five original research papers, recent advances and applications of computational chemogenomics in pharmacological research and drug discovery.

Hu et al. presents the identification of novel inhibitors of a mutant of Isocitrate dehydrogenase (IDH), IDH1-R132C, an oncogenic metabolic enzyme. The inhibitors were identified by means of docking-based virtual screening of a commercial synthetic library with 1.5 million compounds and cellular inhibition assays. The most promising compound (with the identifier T001-0657) showed high selectivity for cancer cells with the IDH1-R132C mutation and could be further developed as a tool compound to further investigate the biological role of the mutant IDH1-R132C. The authors explored the potential molecular mechanism of the newly identified compound and structural domain of IDH1-R132C with molecular dynamics simulations and free energy calculations. Ghosh et al. discuss the computational characterization of thirty aryl benzoyl hydrazide derivatives with experimental evaluations as inhibitors of the RNA-dependent RNA polymerase enzyme of the H5N1 influenza virus. The authors investigated the structural requirements for antiviral properties of the compounds with 2D-quantitative structure-activity relationship (2D-QSAR), 3D-QSAR, structure-based pharmacophore modeling, molecular docking, and molecular dynamics simulations. Specifically, molecular docking was employed to generate a structure-based pharmacophore mapping. Molecular dynamics was conducted to assess the dynamic stability of the docked ligands at the proposed binding site of the receptor. The binding interactions obtained from the molecular dynamics simulations had a good agreement with the findings of the 2D-QSAR, 3D-QSAR, and pharmacophore modeling. Cofas-Vargas et al. explore the binding of the fungal antibiotic aurovertin, which is an exogenous allosteric inhibitor of the enzyme FOF1-ATP synthase. This enzyme carries out several major regulatory functions in the cell membrane so that its malfunction has been associated with a growing number of human diseases. It is also a promising drug target to combat antibiotic resistance. To characterize the protein-ligand interactions, the authors employed mixed-solvent molecular dynamics, end-point binding free energy calculations, and free energy calculations. The findings of this work helped to provide insights for the structure-based design of novel allosteric drugs targeting FOF1-ATP synthase sites of exogenous inhibitors. Yang et al. identify dodoviscin A as an inhibitor of the extracellular signal-regulated protein kinase 2 (ERK2). Toward this goal, authors conducted docking-based virtual screening of a natural product collection prefiltered with estimated ADMETox properties. As part of the structure-based virtual screening, the authors compared the calculated binding energies of the screening collection with desirable pharmacokinetic characteristics with magnolipin, a known inhibitor of ERK2. The putative binding mode and stability of dodoviscin A with the binding site of ERK2 were explored with molecular dynamics simulations.

Liu et al. introduce the free web-based database of pharmaco-omics for cancer precision medicine (DBPOM). The database provides the reversed and adverse effects of 19,406 small-molecules and drugs and 509 drug combinations on the patients of five cancer types based on the genomics and transcriptomics information of 28 kinds of cell lines and 3078 cancer samples. In their work, the authors also describe a methodology to predict the drug’s efficacy to reverse or enhance their cancer-associated gene expression change. Liu et al. anticipate that the webserver DBPOM will become a valuable platform for drug development, drug mechanism studies and the discovery of new therapies.

In conclusion, the authors, the Research Topic Editors, and the journals of Frontiers in Pharmacology and Frontiers in Drug Discovery anticipate that this Research Topic contributes to illustrate the progress made in the development, validation, and successful applications of *in silico* approaches to pharmacological research and drug discovery. It is expected that the article collection will motivate, inform, and provide direction to the researchers and students in the field.
